# Case report: Multimodality imaging of unusual coronary to pulmonary collaterals in chronic thromboembolic pulmonary hypertension

**DOI:** 10.3389/fcvm.2023.1204736

**Published:** 2023-06-08

**Authors:** Ansh Goyal, Ryan Avery, Michael J. Cuttica, James D. Flaherty, S. Chris Malaisrie, Ruben Mylvaganam

**Affiliations:** ^1^ Department of Cardiac Surgery, Bluhm Cardiovascular Institute, Northwestern University Feinberg School of Medicine and Northwestern Memorial Hospital, Chicago, IL, United States; ^2^ Department of Radiology, Northwestern University Feinberg School of Medicine and Northwestern Memorial Hospital, Chicago, IL, United States; ^3^ Department of Pulmonary and Critical Care, Northwestern University Feinberg School of Medicine and Northwestern Memorial Hospital, Chicago, IL, United States; ^4^ Department of Cardiology, Bluhm Cardiovascular Institute, Northwestern University Feinberg School of Medicine and Northwestern Memorial Hospital, Chicago, IL, United States

**Keywords:** CTEPH, DECT, pulmonary endarterectomy, coronary angiogram, pulmonary angiography, collaterals, chronic thromboembolic pulmonary hypertension

## Abstract

We present unusual coronary-pulmonary collaterals in a 65-year-old CTEPH patient. Perfusion mapping of a dual-energy computed tomography (DECT) study revealed areas of right lung that were minimally perfused despite unilateral occlusion of the right pulmonary artery, leading to the discovery of coronary-pulmonary collaterals *via* invasive coronary angiography. Pulmonary thromboendarterectomy removed the clot en-bloc. Post-surgery DECT and catheterization confirmed restoration of pulmonary arterial circulation and excellent hemodynamic response. Here, suggestion of perfusion to a proximally obstructed lung with DECT helped to document the presence of rarely documented coronary-pulmonary artery collaterals.

## Introduction

Chronic thromboembolic pulmonary hypertension (CTEPH) is classified as group 4 pulmonary hypertension where unresolved thromboembolic disease obstructs the pulmonary artery. The resultant lung ischemia commonly leads to bronchial arterial collateralization thought to lower pulmonary vascular resistance and reduce mortality (1). While CTEPH is frequently studied with CT-PA and ventilation-perfusion scans, dual-energy CT (DECT) scans are an emerging evaluation tool among others including photon counting CT or rapid kV switching dual-energy CT (2–4). DECT depends on dual sensor-based simultaneous computed tomography scans at two different tube voltages with contrast iodine. This allows for subsequent image processing based on material decomposition detected as attenuation differences at different energy levels to calculate perfusion blood volume (PBV) at each voxel and accordingly visualize contrast uptake based on a given color scale. Depending on phase of contrast uptake, perfusion defects are visualized such as in V/Q imaging during the arterial phase, while imaging timed later can reveal perfusion due to collaterals (5). A DECT study is dose neutral when compared to a standard CTA, though some studies have shown some differences depending on employed technique and differences in normalization of image quality, signal to noise ratio and dose length product (6–8). Here, images were acquired with a dual energy-dual source scanner with 2 separate 64-row detectors and image postprocessing was completed using Syngo DE Lung PBV, Siemens Healthcare software, which uses the three-material decomposition theory of iodine, air and soft-tissue to calculate PBV. Ultimately, patients with CTEPH may be candidates for surgical cure with pulmonary thromboendarterectomy based on image-guided assessment and clinical status (9). Th**e** purpose of this case study is to describe utility of DECT-PBV to evaluate pulmonary embolism and its functional consequences as well as illustrate its role for the first time in characterizing the rarely documented coronary-pulmonary collaterals in a unilateral CTEPH patient.

## Case presentation

We present a 65-year-old former 40-pack year smoker with a medical history of chronic obstructive pulmonary disease requiring supplemental oxygen, leading to atrial fibrillation, diastolic heart failure, and chronic thromboembolic pulmonary hypertension (CTEPH) who was referred to our institution for advanced surgical management of CTEPH. On admission, the patient was experiencing increased dyspnea and significant oxygen desaturation, necessitating transfer to our institution for further evaluation and diagnostics.

The patient was in his usual state of health until 4 years prior when he developed right lower extremity swelling and dyspnea and was diagnosed with a deep vein thrombosis (DVT) and pulmonary embolism (PE). Review of these historical records demonstrated extensive pulmonary embolic material beginning in the right main pulmonary artery extending into segmental and subsegmental arteries. He was initiated on warfarin and remained adherent with therapeutic INR's.

Transthoracic echocardiography revealed a normal sized left ventricle with an ejection fraction of 64%. The right ventricle was moderately dilated with decreased systolic function, there was interventricular flattening in both systole and diastole, the right atrium was severely enlarged, and estimated right ventricular systolic pressures were elevated. RV basal diameter was 4.45 cm, RA volume was 94.3 ml, TAPSE was 1.71 cm, and S’ prime was 9.79 cm/s.

**Figure 1:**
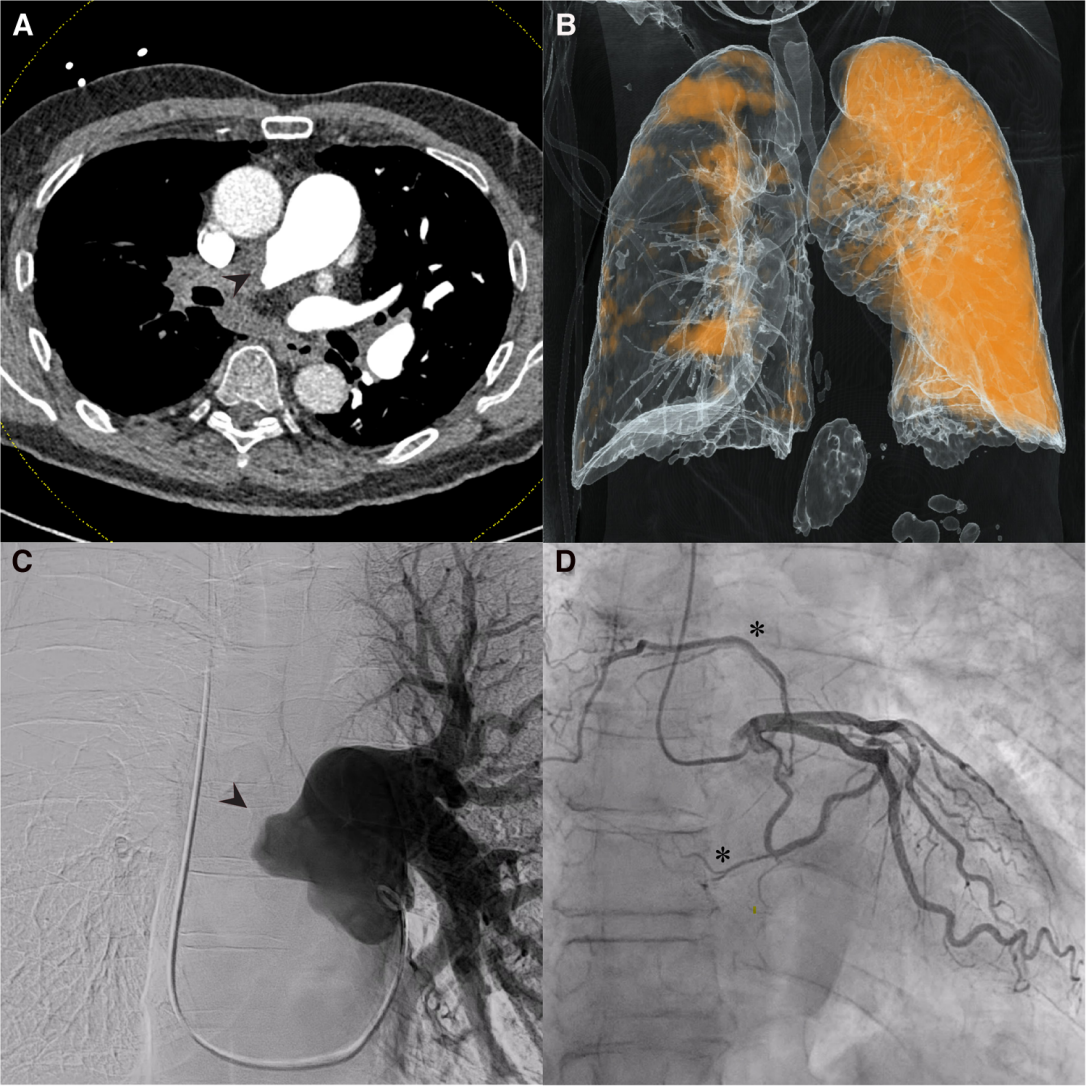
Dual-Energy computed tomography (DECT), pulmonary and coronary angiography. (**A**) Coronal DECT image demonstrating obstruction of right pulmonary artery (arrowhead); (**B**) Perfusion mapping demonstrates limited perfusion of right lung from unknown systemic supply at time of imaging. (**C**) Pulmonary angiography confirmed complete obstruction of right pulmonary circulation (arrowhead); (**D**) Coronary angiography revealed multiple systemic coronary-pulmonary collaterals from the left circumflex artery (asterisks).

Dual-energy computed tomography (DECT) redemonstrated unilateral complete occlusion of right pulmonary arterial circulation with thrombus propagation into the proximal right pulmonary artery. No filling defects in the left pulmonary arterial system were visualized. Despite the apparent complete obstruction of the right pulmonary artery, perfused blood volume (PBV) mapping of the dual energy CT study revealed areas of the right lung that were still minimally perfused (Figure 1 A,B).

Pulmonary angiography with left and right heart catheterization was undertaken for surgical candidacy and planning. Right heart catheterization revealed a right atrial pressure of 4mmHg, mean pulmonary artery pressure of 40mmHg, pulmonary wedge pressure of 4mmHg, cardiac output of 3.5, cardiac index of 1.8 and pulmonary vascular resistance of 10.3 wood units. Pulmonary angiography clearly demonstrated complete obstruction of the right pulmonary artery (Figure 1C). Coronary angiography revealed the presence of coronary to pulmonary collaterals traveling to the pulmonary circulation from the left circumflex artery (Figure 1D). An aortogram was not clinically indicated as systemic collateral flow has been classically described and is not routinely investigated in CTEPH. With the delayed timing of the DECT images, it is likely that the perfusion mapping in the right hemithorax represents collateral flow through the coronary-pulmonary collaterals seen on the coronary angiography, bronchial collaterals, or both. There was no evidence the patient experienced coronary steal syndrome or angina given the presence of these coronary-pulmonary collaterals.

**Figure 2:**
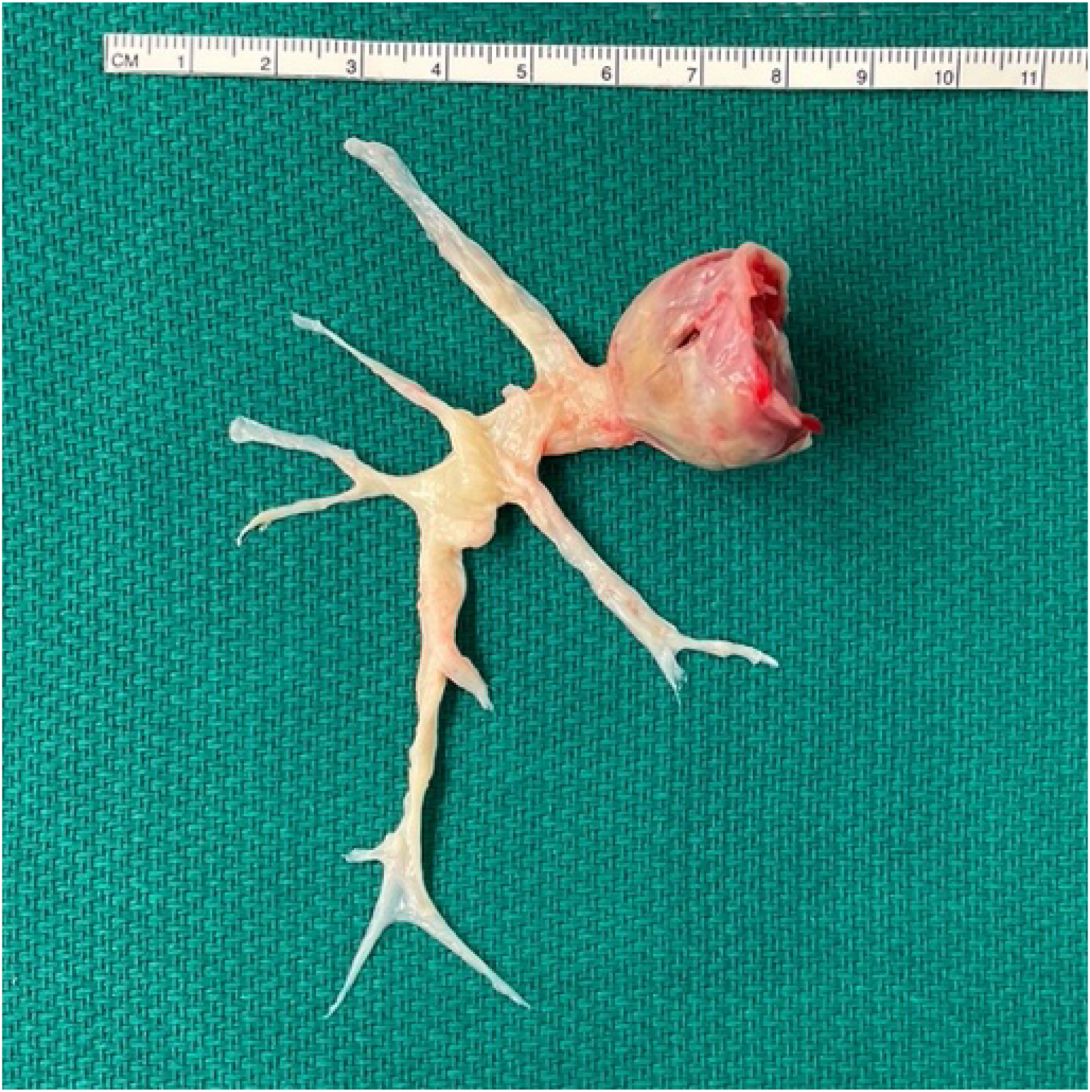
Surgical specimen. Entire clot removed en-bloc *via* thromboendarterectomy.

The patient underwent successful pulmonary thromboendarterectomy, Cox-Maze 3, and left atrial appendage occlusion without complication. Briefly, a primary median sternotomy was performed, bypass was initiated, and aortic cross clamp was applied. The right pulmonary artery was opened, and the entire clot was removed *en bloc* from all 10 segments of the right pulmonary artery under hypothermic circulatory arrest (Figure 2). The left pulmonary artery was inspected and found to have no significant clot consistent with pre-operative imaging. After bypass flows were resumed, an atrial clip was placed at the base left atrial appendage externally and atriotomies were performed to complete the Cox-Maze 3 procedure.

**Figure 3:**
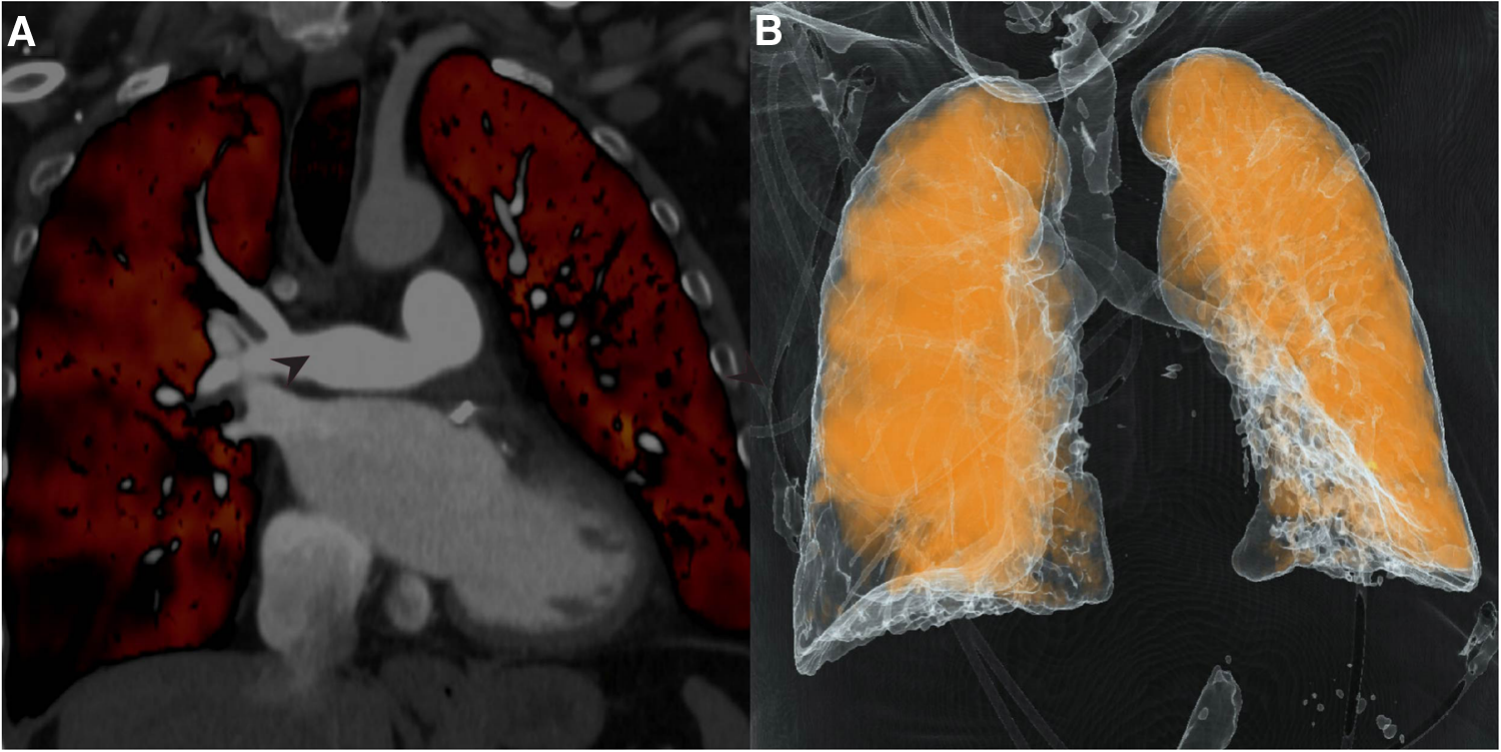
Dual-Energy computed tomography (DECT). (**A**) Repeat DECT demonstrates patent pulmonary artery post-thromboendarterectomy; (**B**) Perfusion mapping reveals significantly improved perfusion of right lung.

Post-surgery DECT confirmed patency of the right pulmonary artery and restoration of segmental and subsegmental pulmonary arterial circulation (Figure 3A,B). Pathology from the cast was negative for malignancy. Catheterization revealed an excellent hemodynamic response to surgery with a right atrial pressure of 3 mmHg (delta −1 mmHg), mean pulmonary artery pressure of 22 mmHg (delta −18 mmHg), cardiac output of 3.9 (delta +0.4), cardiac index of 2.0 (delta +0.2) and pulmonary vascular resistance of 4.9 wood units (delta −5.4 wood units). Repeat echocardiography revealed RV basal diameter of 3.84 cm (delta −0.61 cm), RA volume of 52.5 ml (delta −41.8 ml), TAPSE of 0.84 cm (delta −0.87 cm), and S’ of 7.87 cm/s (delta −1.92 cm/s).

## Discussion

**Figure 4:**
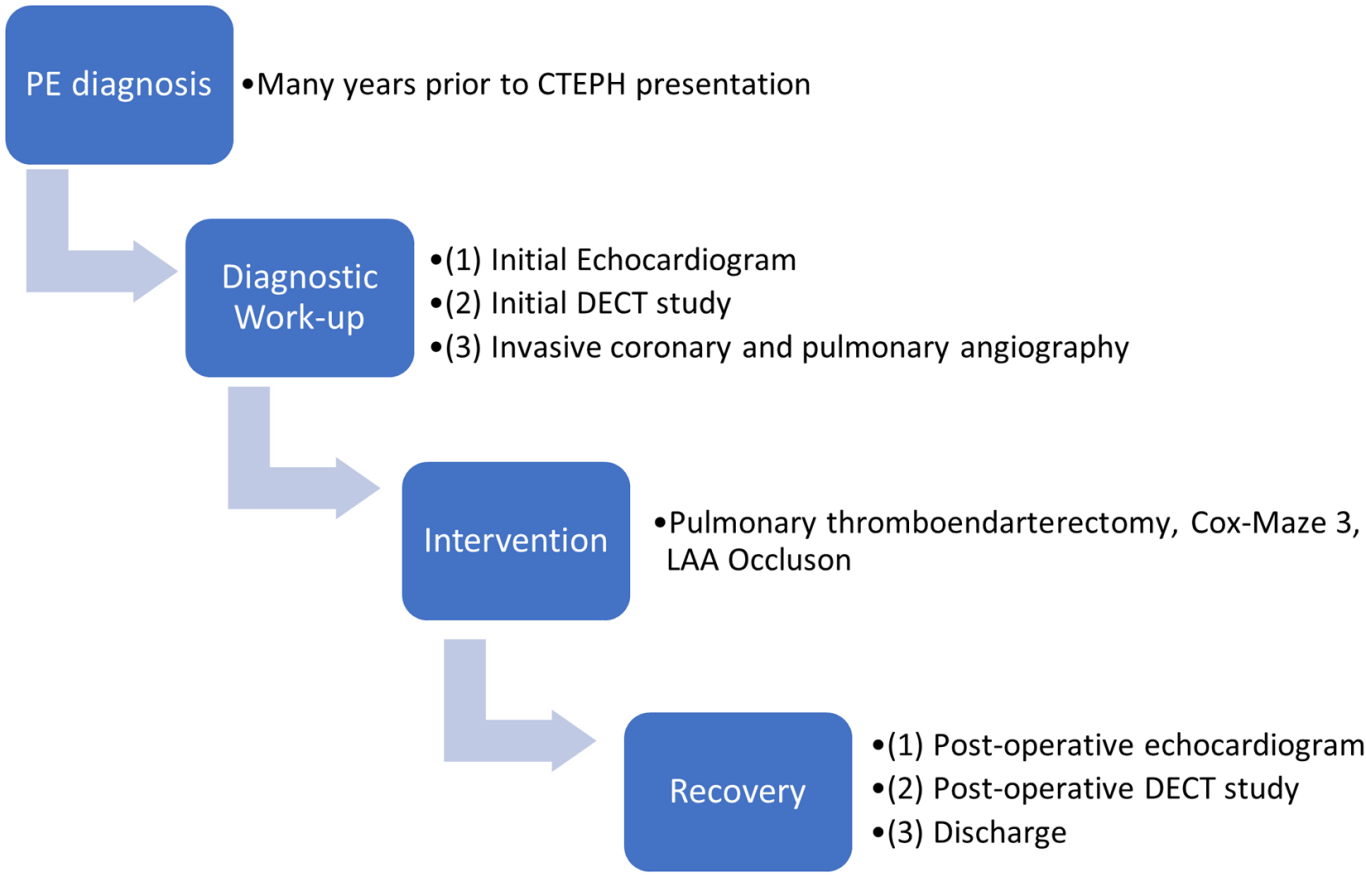
Timeline of events associated with the care episode.

We highlight that DECT-derived PBV mapping is a uniquely powerful tool to characterize the pulmonary vasculature, and to some extent the systemic vasculature, of patients and should receive more widespread consideration for evaluation of CTEPH because it allows for simultaneous assessment of pulmonary vessel obstruction and arterial perfusion to confirm diagnoses and guide clinical decision-making (Figure 4). First, significant focal perfusion to a proximally obstructed lung was revealed, eventually leading to a diagnosis of unilateral CTEPH. Unilateral CTEPH is independently a rarely discovered entity, seemingly documented only in case reports in patients with underlying thrombophilic disorders (10, 11). Whether the unilateral nature of this patient's disease contributed in some manner to the development of the coronary-pulmonary collaterals in this patient without a thrombophilia is unknown. Second, the DECT study revealed greater than expected perfusion to the obstructed lung. As a result, when invasive angiography was pursued, special attention was paid to elucidate the source of collateral flow, leading to the discovery of the rarely documented coronary-pulmonary artery collaterals. The physiological and molecular mechanisms underpinning angiogenesis and development of coronary-pulmonary collaterals are unclear. The contribution of systemic to pulmonary collaterals and the two-compartment model of pathophysiology in CTEPH remains to be elucidated. Thus far, while documentation of these collaterals in CTEPH patients has remained limited, research has suggested that the prevalence of coronary-pulmonary collaterals in CTEPH may be higher than previously thought (12–15). Bronchopulmonary collaterals in CTEPH have been more commonly documented and are suggested to promote bronchial dilation, lower post-operative pulmonary vascular resistance, and ultimately reduce post-operative mortality (1). Ultimately, it may be that bronchial collateralization contributed to some of the perfusion findings; however, the location of the discovered coronary-pulmonary collaterals vs. the PBV/perfusion map suggests they were a significant contributor as well. These coronary-pulmonary collaterals may share pathophysiology similar to bronchial-pulmonary collaterals, decrease pulmonary arterial resistance, and may also develop due to hypoxic factors. Our institution has adopted a delayed phase imaging protocol with the use of DECT to help understand and identify collateral flow.

## Data Availability

The original contributions presented in the study are included in the article, further inquiries can be directed to the corresponding author.

## References

[1] Delcroix M Vonk Noordegraaf A Fadel E Lang I Simonneau G Naeije R. Vascular and right ventricular remodelling in chronic thromboembolic pulmonary hypertension. Eur Respir J. (2013) 41(1):224–32. 10.1183/09031936.00047712 22903956

[2] Heinrich M Uder M Tscholl D Grgic A Kramann B Schäfers HJ. CT Scan findings in chronic thromboembolic pulmonary hypertension: predictors of hemodynamic improvement after pulmonary thromboendarterectomy. Chest. (2005) 127(5):1606–13. 10.1378/chest.127.5.1606 15888835

[3] Hong YJ Shim J Lee SM Im DJ Hur J. Dual-Energy CT for pulmonary embolism: current and evolving clinical applications. Korean J Radiol. (2021) 22(9):1555–68. 10.3348/kjr.2020.1512 34448383 PMC8390816

[4] Abdellatif W Ebada MA Alkanj S Negida A Murray N Khosa F Diagnostic accuracy of dual-energy CT in detection of acute pulmonary embolism: a systematic review and meta-analysis. Can Assoc Radiol J. (2021) 72(2):285–92. 10.1177/0846537120902062 32103682

[5] Petritsch B Kosmala A Gassenmaier T Weng AM Veldhoen S Kunz AS Diagnosis of pulmonary artery embolism: comparison of single-source CT and 3rd generation dual-source CT using a dual-energy protocol regarding image quality and radiation dose. Rofo. (2017) 189(6):527–36. Diagnostik der akuten Lungenarterienembolie: Vergleich von Single-Source CT und Dritt-Generation Dual-Source CT unter Einsatz eines Dual-Energy Protokolls—Bildqualität und Strahlenexposition. 10.1055/s-0043-103089 28445908

[6] Krauss B Grant KL Schmidt BT Flohr TG. The importance of spectral separation: an assessment of dual-energy spectral separation for quantitative ability and dose efficiency. Invest Radiol. (2015) 50(2):114–8. 10.1097/rli.0000000000000109 25373305

[7] Faby S Kuchenbecker S Sawall S Simons D Schlemmer HP Lell M Performance of today's Dual energy CT and future multi energy CT in virtual non-contrast imaging and in iodine quantification: a simulation study. Med Phys. (2015) 42(7):4349–66. 10.1118/1.4922654 26133632

[8] Henzler T Fink C Schoenberg SO Schoepf UJ. Dual-energy CT: radiation dose aspects. AJR Am J Roentgenol. (2012) 199(5 Suppl):S16–25. 10.2214/ajr.12.9210 23097163

[9] Kim NH. Assessment of operability in chronic thromboembolic pulmonary hypertension. Proc Am Thorac Soc. (2006) 3(7):584–8. 10.1513/pats.200605-106LR 16963538

[10] Hon S Channick RN Farber HW. Unilateral chronic thromboembolic pulmonary disease: a mimic of pulmonary artery agenesis. Am J Respir Crit Care Med. (2020) 201(10):e74–5. 10.1164/rccm.201905-0997IM 31899659

[11] Laczika K Lang IM Quehenberger P Mannhalter C Muhm M Klepetko W Unilateral chronic thromboembolic pulmonary disease associated with combined inherited thrombophilia. Chest. (2002) 121(1):286–9. 10.1378/chest.121.1.286 11796466

[12] Lee NS Auger WR Pretorius V Blanchard DG Daniels LB. Coronary artery-to-pulmonary artery collaterals in chronic thromboembolic pulmonary hypertension. Circ Cardiovasc Imaging. (2014) 7(6):962–6. 10.1161/circimaging.114.002368 25406199

[13] Lee NS Blanchard DG Knowlton KU McDivit AM Pretorius V Madani MM Prevalence of coronary artery-pulmonary artery collaterals in patients with chronic thromboembolic pulmonary hypertension. Pulm Circ. (2015) 5(2):313–21. 10.1086/681225 26064456 PMC4449242

[14] Haghbayan H Coomes EA Gunaratne K. A man in his 40s with coronary-to-pulmonary artery collateralizations. JAMA Cardiol. (2020) 5(3):356. 10.1001/jamacardio.2019.5289 31940004

[15] Kim MS Jung JI Chun HJ. Coronary to pulmonary artery fistula: morphologic features at multidetector CT. Int J Cardiovasc Imaging. (2010) 26(Suppl 2):273–80. 10.1007/s10554-010-9711-3 20878252

